# Data Assimilation to extract Soil Moisture Information from SMAP Observations

**DOI:** 10.3390/rs9111179

**Published:** 2017-11-17

**Authors:** Jana Kolassa, Rolf H. Reichle, Qing Liu, Michael Cosh, David D. Bosch, Todd G. Caldwell, Andreas Colliander, Chandra Holifield Collins, Thomas J. Jackson, Stan J. Livingston, Mahta Moghaddam, Patrick J. Starks

**Affiliations:** 1 Universities Space Research Association, Columbia, MD; 2 Global Modeling and Assimilation Office, NASA Goddard Space Flight Center, Greenbelt, MD; 3 Science Systems and Applications Inc., Lanham, MD; 4 Bureau of Economic Geology, the University of Texas at Austin, Austin, TX; 5 Jet Propulsion Laboratory, California Institute of Technology, Pasadena, CA; 6 USDA ARS Hydrology and Remote Sensing Laboratory, Beltsville, MD; 7 USDA ARS Southwest Watershed Research Center, Tucson, AZ; 8 University of Southern California, Los Angeles, CA; 9 USDA ARS Grazinglands Research Laboratory, El Reno, OK; 10 USDA ARS Southeast Watershed Research Center, Tifton, GA; 11 USDA ARS National Soil Erosion Research Laboratory, West Lafayette, IN

**Keywords:** data assimilation, SMAP soil moisture, neural networks, bias correction

## Abstract

This study compares different methods to extract soil moisture information through the assimilation of Soil Moisture Active Passive (SMAP) observations. Neural Network (NN) and physically-based SMAP soil moisture retrievals were assimilated into the NASA Catchment model over the contiguous United States for April 2015 to March 2017. By construction, the NN retrievals are consistent with the global climatology of the Catchment model soil moisture. Assimilating the NN retrievals without further bias correction improved the surface and root zone correlations against in situ measurements from 14 SMAP core validation sites (CVS) by 0.12 and 0.16, respectively, over the model-only skill and reduced the surface and root zone ubRMSE by 0.005 m^3^ m^−3^ and 0.001 m^3^ m^−3^, respectively. The assimilation reduced the average absolute surface bias against the CVS measurements by 0.009 m^3^ m^−3^, but increased the root zone bias by 0.014 m^3^ m^−3^. Assimilating the NN retrievals after a localized bias correction yielded slightly lower surface correlation and ubRMSE improvements, but generally the skill differences were small. The assimilation of the physically-based SMAP Level-2 passive soil moisture retrievals using a global bias correction yielded similar skill improvements, as did the direct assimilation of locally bias-corrected SMAP brightness temperatures within the SMAP Level-4 soil moisture algorithm. The results show that global bias correction methods may be able to extract more independent information from SMAP observations compared to local bias correction methods, but without accurate quality control and observation error characterization they are also more vulnerable to adverse effects from retrieval errors related to uncertainties in the retrieval inputs and algorithm. Furthermore, the results show that using global bias correction approaches without a simultaneous re-calibration of the land model processes can lead to a skill degradation in other land surface variables.

## 1. Introduction

The importance of soil moisture in hydrological and land surface boundary layer processes has long been recognized (e.g. *Seneviratne et al.* [1], *Bateni and Entekhabi* [2], *Assouline* [3], *Jung et al.* [4]), and the need for high quality soil moisture observations to enhance our understanding of these processes has been identified [5]. Direct observations of soil moisture can be obtained with in situ sensors, but these are constrained to point-scale measurements at a limited number of locations.

In contrast, satellite instruments are able to observe soil moisture globally with a local revisit time of 2–3 days. In particular L-band (1.4 GHz) microwave radiometers have a high soil moisture sensitivity and are able to penetrate the top 5 cm of the soil in sparsely to moderately vegetated areas [6,7]. Two passive L-band satellite missions have been launched in recent years, the European Space Agency’s Soil Moisture and Ocean Salinity (SMOS) mission in 2009 [8] and the National Aeronautics and Space Administration’s Soil Moisture Active Passive (SMAP) mission in 2015 [7]. Soil moisture retrieval products from SMOS and SMAP have been shown to have high skill in capturing soil moisture variations [9,10], however, many applications require observations of the complete soil moisture profile and with finer spatial and temporal resolutions than those of SMOS and SMAP.

Data assimilation (DA) can be used to interpolate and extrapolate the satellite observations by merging them with information from a dynamic land surface model. This generates higher horizontal resolution estimates of the full soil moisture profile with complete spatio-temporal coverage and often with a higher skill than that of the model or satellite observations alone [11–18].

The specific soil moisture skill improvements that can be obtained from an assimilation depend on the quality of the assimilated observations, the amount of complementary/novel information they provide, and the efficiency with which the DA system is able to extract this information. The latter is contingent on the specifics of the DA system, including (but not limited to) the type of observation assimilated (raw brightness temperatures (Tb) vs. soil moisture retrievals), the assimilation algorithm, the observation and model error estimates, and the bias correction scheme. Ultimately, the optimal choice for each factor and their combination depends on the specific application and a simultaneous comparison of all possible options is not trivial. Nevertheless, several studies have explored options for the individual factors in the context of DA for soil moisture estimation. For example, *De Lannoy and Reichle* [17] compared the assimilation of SMOS soil moisture retrievals against the assimilation of (two versions of) SMOS Tbs and showed that in each case different information was extracted from the observations resulting in locally different soil moisture estimates. *Crow and Van den Berg* [19] investigated the use of an independent triple collocation (TC) analysis to generate improved estimates of the model and observation errors. Finally, *Kumar et al.* [20] explored two methods to correct the observation bias, while *De Lannoy et al.* [21] investigated methods to correct the model forecast bias.

One key assumption for most DA algorithms, including the Ensemble Kalman Filer used here, is that all errors are purely random and thus that the observations are unbiased with respect to the model (e.g. *Kalnay* [22] Chapter 5). Realistically, biases in the model forcing data, differences in the soil texture or biases in the Tbs will generally result in biases between the observations and the model. To comply with the assumption of unbiased observations, DA systems typically rescale the observations to the model climatology (generally referred to as ‘bias correction’). One common approach is to match the cumulative distribution function (CDF) of the observations to that of the model estimates at each location [23,24]. Alternatively, *Reichle et al.* [18] rescale the assimilated Tbs such that their seasonally-varying climatology matches that of the simulated Tbs in each location. While such localized bias correction techniques fulfill the requirements of the DA system, they can considerably alter the spatial and temporal patterns of the observation mean and variability, thereby removing some of the independent information provided by the satellite instruments. With the availability of high quality soil moisture retrievals from SMOS and SMAP, it is desirable to retain as much of the independent satellite information as possible.

Our objective in this study is to compare different methods to rescale the observations and identify which approach results in the most efficient assimilation of SMAP soil moisture observations into the NASA Catchment land surface model (CLSM). Specifically, we are interested in the potential of assimilating Neural Network (NN) based retrievals to reduce the need for further bias correction. Recently, *Kolassa et al.* [25] trained a NN on SMAP Tbs and CLSM soil moisture estimates to generate soil moisture retrievals that are, by design, consistent with the global climatology of the model. Here, we assimilate these SMAP NN retrievals without further bias correction and compare the skill of the resulting soil moisture estimates against (1) an assimilation of the SMAP NN retrievals using a standard localized rescaling and (2) an assimilation of the SMAP Level-2 passive soil moisture retrievals using a global rescaling. We additionally compare the skill of the above soil moisture assimilation estimates against that of the SMAP Level-4 soil moisture product, which is based on the assimilation of locally rescaled Tb observations.

## 2. Datasets

### 2.1. SMAP Soil Moisture Products

SMAP was launched in January 2015 and is equipped with an L-band (1.4 GHz) radiometer that observes horizontal and vertical polarization Tbs as well as the 3rd and 4th Stokes’ parameters. Its sun-synchronous, near-circular, polar orbit has equator crossings at 6 AM and 6 PM local time and a revisit time of 2–3 days [7]. Level 1 Tbs have been collected since 31 March, 2015 and are provided on the 36-km resolution Equal-Area Scalable Earth version 2 (EASEv2) grid [26] as daily half-orbit files. Here we use the SMAP NN soil moisture retrieval product [25], the official SMAP Level-2 passive soil moisture retrieval product [10], and the SMAP Level 4 soil moisture analysis [18].

#### 2.1.1. SMAP Neural Network Retrieval Product (SMAP NN)

The details of the SMAP NN retrieval algorithm and product are discussed in *Kolassa et al.* [25]. In this subsection, we briefly summarize the key aspects, following some of their text. The SMAP NN product uses a statistical NN retrieval algorithm to compute surface soil moisture estimates for the 2-year period from April 2015 to March 2017. The data are provided with a 2–3 day resolution and posted on the 36-km resolution EASEv2 grid. The inputs to the retrieval algorithm are brightness temperatures and Stokes’ parameters from the SMAP Level-1C product [27], surface-layer soil temperature estimates from a CLSM simulation (section 3.1), and vegetation water content (VWC) estimated empirically from a MODIS-based NDVI climatology. Only observations from the morning (6 AM) overpass are used in order to minimize observation errors due to Faraday rotation and the difference between the soil and canopy temperatures [7,28]. Soil moisture estimates are computed for times and locations where the soil is unfrozen (GEOS-5 surface temperature is higher than 1°C), the VWC is less than 5 kg m^−2^, and the water fraction of the grid cell is less than 5% according to the GEOS-5 land mask.

Since the NN algorithm is calibrated using CLSM surface soil moisture estimates as the target data, the resulting SMAP NN soil moisture estimates are consistent with the global CLSM climatology, that is, the retrievals match the *global* mean, variability and higher moments of the model estimates. The spatial and temporal patterns of the retrieval product, however, are driven by the satellite input observations (e.g. *Jimenez et al.* [29]). The retrieval errors are estimated through a TC analysis using surface soil moisture retrievals from the Advanced Microwave Scanning Radiometer 2 (AMSR2; [30]) and the Advanced Scatterometer (ASCAT; *Wagner et al.* [31]) as additional inputs [25]. Based on the TC results, the observation error standard deviations for the assimilation are specified as a spatially varying, temporally static error standard deviation map with a global mean value of 0.020 m^3^ m^−3^. For the assimilation experiment with localized bias correction (section 3.2), the observation error standard deviations are rescaled using the ratio of the local (grid cell) model and retrieval soil moisture time series standard deviations. The purpose of this local rescaling is to preserve the relative magnitude of the observations and their errors before and after the local CDF-matching, which matches the observation mean and standard deviation to those of the model.

#### 2.1.2. SMAP Level-2 Passive Retrieval Product (SMAP L2P)

The SMAP Level-2 Passive (L2P) soil moisture estimates are computed from the SMAP radiometer Level-1C Tbs using the physically-based “tau-omega” model [32]. The ancillary input data include surface temperature estimates provided by the quasi-operational GEOS-5 Forward Processing system [33] with a 0.25 ° resolution and VWC estimated from a MODIS-based NDVI climatology using an empirical relationship established from prior observations. No retrieval is performed for frozen soil conditions (fraction of frozen soil based on GEOS-5 surface temperature larger than 5%) and soil moisture estimates are flagged as ‘not recommended’ for dense vegetation (VWC > 5 kg m^−2^) [28]. The soil moisture estimates are provided as daily half-orbit files on the 36-km resolution EASE v2 grid that is also used for the SMAP NN retrieval product.

Here, we used version 4 of the SMAP L2P ‘baseline’ retrieval product that is based on SMAP vertical polarization Tbs [34]. We assimilated only data points from the morning (6 AM) overpasses and for which the retrieval quality flag was set to ‘recommended’ (indicating unfrozen soils and a VWC below 5 kg m^−2^). Based on a TC analysis [25], the observation error standard deviations for the assimilation were specified as a static error standard deviation map with a global mean of 0.030 m^3^ m^−3^. For the assimilation of the L2P retrievals with global bias correction, the error standard deviations were rescaled using the ratio of the global model and observation standard deviations (computed over all times and locations).

#### 2.1.3. SMAP Level-4 Soil Moisture Analysis (SMAP L4_SM)

The SMAP L4_SM data product is generated by assimilating SMAP Level-1C Tb anomalies into the CLSM using the DA system discussed in section 3.1 combined with a tau-omega radiative transfer model [18,35]. SMAP Tbs with an ‘acceptable’ quality flag - as defined in [36] - are assimilated when the model does not indicate active precipitation, frozen soil or snow cover. L-band brightness temperatures generally exhibit a spatially and temporally varying bias with respect to the CLSM (see e.g., Figure 2 of *De Lannoy and Reichle* [16]). To account for this, the SMAP observations are locally rescaled prior to the assimilation such that their seasonally-varying climatology (i.e., their long-term mean seasonal cycle) matches that of the model. SMAP L4_SM estimates of the volumetric surface (0–5 cm) and root zone (0–100 cm) soil moisture are available globally on the 9-km EASEv2 grid with a 3-hourly resolution.

Here, we extracted surface and root zone soil moisture estimates from the Version 2 L4_SM data [18,37] for our study period and study domain (section 3.2).

### 2.2. In Situ Data

We evaluate the model soil moisture estimates against in situ soil moisture measurements from the SMAP core validation sites and two sparse networks.

#### 2.2.1. Core Validation Site Measurements

The SMAP core validation sites (CVS) are a diverse collection of calibration and validation sites across different watersheds that use dense arrays of soil moisture sensors distributed over so-called reference pixels at 3-km, 9-km and 36-km to represent the spatial scales of the different SMAP products [38]. The measurements from sensors within each reference pixel are combined into an area-weighted average using weights based on Voronoi polygons [38] to yield one in situ soil moisture time series per reference pixel that is representative of a SMAP grid cell. Here we use reference pixels at the 9-km scale (matching the resolution of the DA experiments) from a subset of CVS located in the contiguous United States. This includes 14 reference pixels (from 8 different watersheds) with surface soil moisture measurements and 8 reference pixels (from 5 different watersheds) that additionally provide root zone soil moisture measurements. These sites span a range of different climatic conditions, land cover and land use types (Table 1).

#### 2.2.2. Sparse Network Measurements

We also evaluate the soil moisture estimates against in situ measurements from the Soil Climate Analysis Network (SCAN; [39]) and the US Climate Reference Network (USCRN; [40,41]). Unlike the CVS, these ‘sparse’ networks typically only have one sensor within each 9-km model grid cell and are not necessarily representative of the grid-cell scale soil moisture estimated by the model. However, the sparse networks span a more varied range of climatic conditions and land cover types than the CVS. We use SCAN and USCRN measurements at 5 cm depth to validate the surface soil moisture from the assimilation. The root zone soil moisture estimates are evaluated against an average of the in situ measurements for the 0–100 cm layer with each measurement weighted by the vertical extent of the represented layer. The SCAN and USCRN data were subjected to an extensive quality control process as detailed, for example, in *De Lannoy et al.* [42] and Appendix C of *Reichle et al.* [43]. After quality control, 181 stations were used from SCAN and 138 from USCRN.

## 3. Data Assimilation System and Experiments

### 3.1. Model and Data Assimilation System

The data assimilation experiments are performed using the CLSM driven with surface meteorological forcing data at 0.25 ° resolution provided by the GEOS-5 Forward Processing system [33]. The precipitation forcing data are corrected using global gauge-based observations from the NOAA Climate Prediction Center Unified (CPCU) product, scaled to the Global Precipitation Climatology Project (GPCP) v2.2 pentad precipitation product climatology [44,45]. The GEOS-5 background precipitation is also scaled to the GPCP v2.2 climatology.

The diagnostics used here to analyze the assimilation results are the surface (0–5cm) and root zone (0–100cm) soil moisture, as well as the land evaporation and the overland runoff. Two different configurations of the model are used in this study: (1) the Nature Run v4 (NRv4) configuration used to generate the L4_SM product and (2) the Nature Run v5 (NRv5) configuration used for the SMAP soil moisture assimilation experiments presented here. The main differences between the two configurations include an updated correction of the precipitation forcing data, an updated vegetation height dataset as well as revised parameterizations of the heat capacity, the minimum snow water equivalent and the turbulent roughness length. The DA system was run over the contiguous United States from April 2015 to March 2017 producing 3-hourly analyses on the 9-km resolution EASEv2 grid.

The assimilation was performed using an Ensemble Kalman Filter including non-zero horizontal correlations in the observation and model errors in order to distribute the observed information to nearby model grid cells (3D Ensemble Kalman Filter) [16,46]. This setup essentially uses the model information to downscale the 36-km SMAP observations to the 9-km model resolution. To translate the model state into surface soil moisture estimates with the same spatial support as the observations, the observation operator computes the spatial convolution of the model estimates with a two-dimensional Gaussian function that contains 50% of the signal within a circle with a radius of 20 km [17]. Observation error maps were estimated using a TC analysis (section 2) and the spatial correlation between the observation errors was assumed to follow a Gaussian distribution with a 0.25° length scale in all directions. Following the SMAP L4_SM setup, an ensemble of 24 members was used here. Moreover, model error correlations were localized to a radius of 1.25 ° (by reducing their value to zero beyond this radius) to avoid spurious spatial correlations as a result of the limited ensemble size [18,47]. The perturbations to the meteorological forcing and model prognostic variables follow the Version 2 L4_SM system [18] and are summarized in Table 2.

### 3.2. Data Assimilation Experiments

Several data assimilation experiments were performed for April 2015 to March 2017 over the contiguous United States (CONUS), each with a different method to address bias between the observations and corresponding model forecasts. All experiments used the modeling and DA system introduced in section 3.1. Table 3 summarizes the main characteristics of all assimilation experiments and section 3.3 discusses limitations associated with each experiment.

#### Open Loop

For the open loop (OL) experiment, the model is run for the study period without assimilating any SMAP observations. The OL represents a baseline for the model skill against which potential skill improvements from the assimilation of SMAP observations are measured. The OL for the soil moisture assimilation experiments is generated using the NRv5 configuration, whereas the OL-L4 for the L4_SM system is generated using the NRv4 configuration (section 3.1).

#### SMAP NN retrieval assimilation without bias correction (DA-NN)

In the DA-NN experiment, the NN retrievals are assimilated without further bias correction. By design, the NN retrievals are consistent with the global climatology of the model. The purpose of this experiment is thus to test whether the NN approach is sufficient to account for the systematic bias (related to factors other than disagreements about the soil moisture state) between the model and observations and thus reduce the need for further rescaling that would remove some of the independent satellite information.

#### SMAP NN retrieval assimilation with local CDF-matching (DA-NN-lCDF)

In the DA-NN-lCDF experiment, the NN retrievals are assimilated after applying a local CDF-matching that imposes the model’s mean, variability and higher moments on the observations separately for each grid cell. To compute the CDF-matching statistics, we apply a spatial sampling with a 1.25° moving window to mitigate the effect of the relatively short study period [23]. The purpose of this experiment is to compare the assimilation using a local (grid cell level) rescaling in DA-NN-lCDF with the global rescaling implicit in the DA-NN experiment.

#### SMAP L2P retrieval assimilation with global CDF-matching (DA-L2P-gCDF)

In the DA-L2P-gCDF experiment, the L2P retrievals are assimilated after applying a global CDF-matching of the satellite soil moisture retrievals to the model estimates. The purpose of this experiment is to (1) compare the impact of the different retrieval algorithms and (2) assess whether applying a global CDF-matching to an existing retrieval product results in a different soil moisture skill than assimilating soil moisture estimates that are by design consistent with the global model climatology.

#### SMAP Level 4 brightness temperature assimilation product (DA-L4)

The SMAP L4_SM product is generated by assimilating SMAP Tb observations (section 2.1.3) and is included here to relate the skill of the above soil moisture assimilation experiments to the skill that can be obtained from a Tb assimilation (bearing in mind that a local rescaling of the Tbs is applied (section 2.1.3)).

### 3.3. Limitations of the DA experiments

In the DA-NN and DA-L2P-gCDF experiments, the soil moisture observations are globally matched to the climatology of the modeled soil moisture. However, local biases and differences in the local variability are retained (see e.g. Figure 3 of *Kolassa et al.* [25]). These can provide very valuable information on missing processes in the model (for example processes related to agricultural practices) or unrealistic process parameterizations. However, from a DA perspective, the retention of local biases violates the assumptions of the DA system, which is designed to deal with random rather than systematic errors. The experiments conducted here investigate whether - in practice - the benefit of retaining more of the independent satellite information can outweigh the adverse effects of violating the DA assumptions. This includes investigating the effect on the modeled soil moisture skill, but also the impact on related variables, such as evaporation or runoff estimates.

Another concern is the possible non-orthogonality of the observation and model errors as a result of the soil temperature information that is shared between the SMAP retrievals and the model. This issue might be exacerbated by the fact that for the global bias correction approaches, the dynamic range of the model and observations will not necessarily match locally and would represent another violation of the DA system assumptions.

Finally, the assimilation and validation periods here include the NN training period (April 2015 - March 2016), which violates the DA assumption of uncorrelated model and observation errors. Owing to the relatively short SMAP record to date, further investigation of this issue must be left for future study.

### 3.4. Evaluation

We compare the different soil moisture assimilation experiments in terms of (1) the statistics of the modeled soil moisture estimates, (2) the soil moisture estimate skill against in situ soil moisture measurements, (3) the consistency of the specified model and observation error statistics with the actual errors and (4) the impact on model fields related to soil moisture.

#### 3.4.1. Soil moisture statistics

To assess the impact of the assimilation on the climatology of the soil moisture estimates, we compare the statistics of the soil moisture fields generated with the assimilation experiments against those generated with the OL. The difference between the mean soil moisture fields highlights areas that experience a general wetting or drying as a result of the assimilation, whereas the difference of the mean soil moisture standard deviations assesses to what extent the assimilation of SMAP observations introduces (or removes) variability in the modeled soil moisture fields. The soil moisture mean values and standard deviations are computed using all model estimates, including times and locations when no SMAP observations were assimilated.

#### 3.4.2. Evaluation against in situ measurements

The soil moisture estimates from each assimilation experiment are evaluated against in situ measurements using the correlation (*R*), absolute bias (|*b*|) and unbiased root mean square error *(ubRMSE).* The metrics are computed using all simulated soil moisture estimates, including time instances when no SMAP observations were assimilated. The correlation is computed as the Pearson correlation coefficient of the modeled and in situ soil moisture time series in each location and quantifies the skill in capturing soil moisture temporal variations across all time scales. The absolute bias is computed as the absolute value of the mean difference between the in situ and modeled soil moisture time series in each location. We use the absolute bias to better compare skill improvements and to avoid the effect of bias compensation when computing mean metrics. The ubRMSE is calculated to estimate errors in the soil moisture variability and is computed as the RMSE between the modeled and in situ soil moisture time series after removing their respective long-term mean values.

To assess the statistical significance of differences in the experiment evaluation metrics, we also estimate their 95% confidence intervals using the Student’s T-test for the correlation and bias, and a chi-square test for the ubRMSE. All metrics and their confidence intervals are estimated accounting for auto-correlation in the soil moisture time series.

When computing average metrics (across all reference pixels for the CVS and across all networks for the sparse networks), we use a k-means clustering approach with a maximum cluster extent of 1° to avoid the dominance of regions with a high sensor density and to ensure realistic confidence intervals [16].

#### 3.4.3. Assimilation diagnostics

The relative impact of the model forecasts and observations on the soil moisture estimates depends on the specified model and observation error statistics. To assess the consistency of the error characterizations in our experiments with the actual model and observation errors, we analyze the standard deviation of the normalized observation-minus-forecast residuals (or ‘innovations’), which are computed as $\frac{\left(O-F\right)}{\sqrt{var\left(e_{O}\right)+var\left(e_{F}\right)}},$ where *O* and *F* are the observations and forecast estimates, and

$var\left(e_{O}\right)\;\text{and}\;var\left(e_{F}\right)$ are the assumed observation and forecast error variances as prescribed $\left(var\left(e_{O}\right)\right)$ or diagnosed from the ensemble $\left(var\left(e_{F}\right)\right)$ [18]. In a well-calibrated DA system, with correctly specified model and observation error statistics, this metric should be close to one. Values greater than one indicate that the DA system underestimates the actual errors, and values less than one indicate that the errors are overestimated. The standard deviation of the normalized observation-forecast differences is computed for times and locations when SMAP observations were assimilated.

#### 3.4.4. Impact on related model fields

The assimilation of soil moisture estimates with a local bias could adversely affect model fields related to soil moisture, despite a potential improvement of the soil moisture estimates themselves (section 3.3). To investigate this possibility, we also analyze changes in the mean land evaporation and overland runoff resulting from the assimilation of SMAP observations. The analysis of the evaporation and runoff is qualitative, since no reliable reference data were available for our study period.

## 4. Results and Discussion

### 4.1. Assimilation with global vs. local bias correction

First, we compare the assimilation of the NN retrievals without further bias correction (DA-NN) to the assimilation of the same retrievals using a standard local CDF-matching bias correction (DA-NN-lCDF).

#### 4.1.1. Mean soil moisture statistics

**Figure 1.**
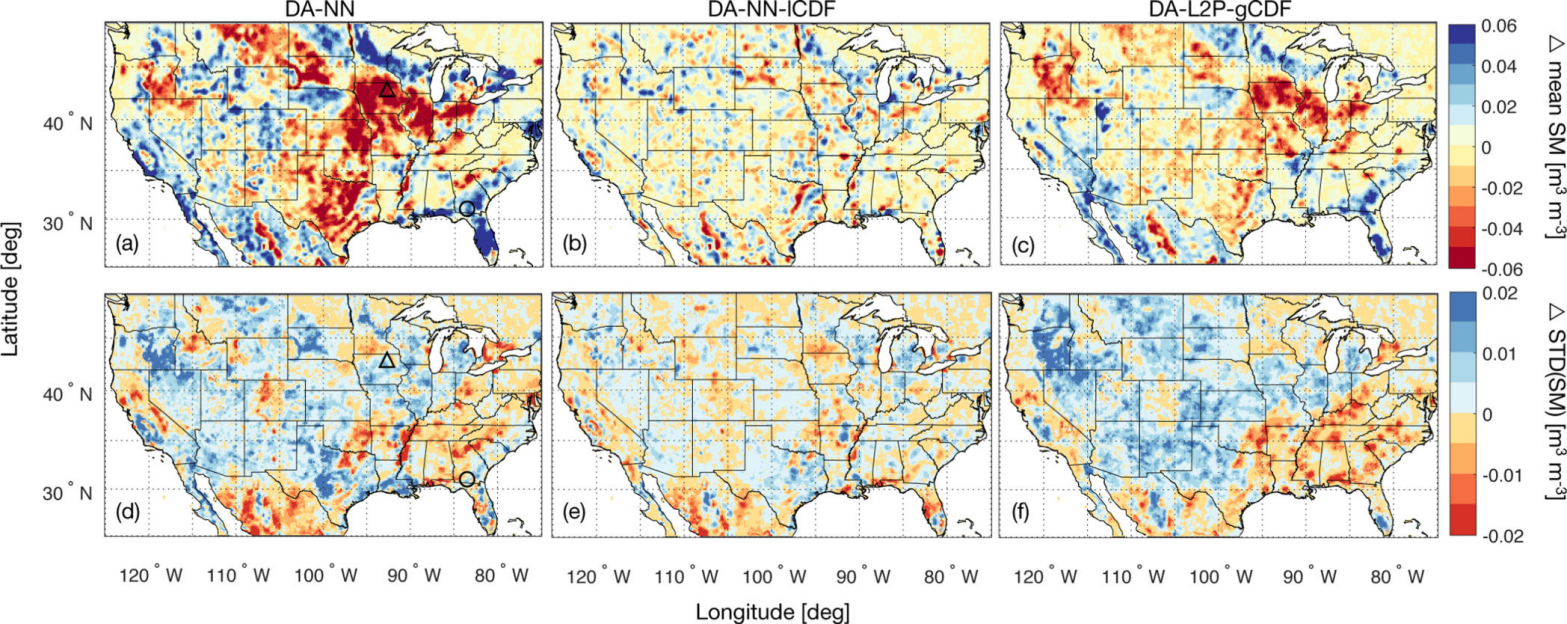
Average soil moisture difference - computed as DA minus OL for the period April 2015 to March 2017 - for the (a) DA-NN, (b) DA-NN-lCDF and (c) DA-L2P-gCDF experiments. Red colors indicate that the assimilation decreases the mean soil moisture with respect to the OL. Panels (d)-(f) are the same, but for the difference of the standard deviation with respect to the OL. Red colors indicate that the assimilation decreases the variability relative to the OL. Panels (a) and (d) also show the location of the South Fork (triangle) and Little River (circle) watersheds discussed in the text.

In the DA-NN experiment, the retention of local biases between the model and observations results in 2-year mean soil moisture estimates that show distinct spatial differences (defined as DA-NN minus OL) with respect to the model (Figure 1 (a)). For example, DA-NN exhibits drier conditions in the predominantly agricultural areas of the Midwest and parts of the Northwest (eastern Montana, eastern Oregon and the Dakotas). In these regions, SMAP observes the effects of agricultural practices (e.g., tile drainage or tillage) that are not represented in the model (see e.g., *He et al.* [48]). For the agricultural areas subject to irrigation, these somewhat counter-intuitive results reflect the dry bias of the SMAP retrievals relative to the model (see e.g., Figure 4(d) in *Kolassa et al.* [25]). In areas with extensive tile drainage, such as large parts of Iowa, the results reflect the expected behavior. Additionally, the spatial patterns of the DA-NN soil moisture estimates depend on the SMAP brightness temperatures as well as the ancillary retrieval inputs and are thus not purely observational features. The local bias correction applied in the DA-NN-lCDF experiment removes systematic differences between the model and the observations prior to the assimilation and - by design - results in mean soil moisture differences without strong spatial features (Figure 1 (b)).

Differences in the soil moisture variability between DA-NN and OL (Figure 1 (d)) appear to be related to the soil moisture mean state and seasonal variability in a region. In humid regions with a more pronounced seasonal cycle, such as parts of the Eastern US, Northern Mexico or the California Central Valley, DA-NN decreases the soil moisture variability with respect to the OL. The reduced variability is possibly an artifact of the retrievals’ reduced soil moisture sensitivity in regions that are more humid and more densely vegetated. One exception to this behavior is the corn belt, where DA-NN increases the soil moisture variability with respect to the OL. Here the NN retrievals capture the effects of agricultural practices that are not represented in the model and that tend to increase the soil moisture variability. The variability differences between the DA-NN-lCDF and the OL (Figure 1 (e)) are generally small and have less distinct spatial features than those observed for the DA-NN experiment, as expected given the local scaling applied to the observations in the DA-NN-lCDF.

#### 4.1.2. Evaluation against in situ measurements

**Figure 2.**
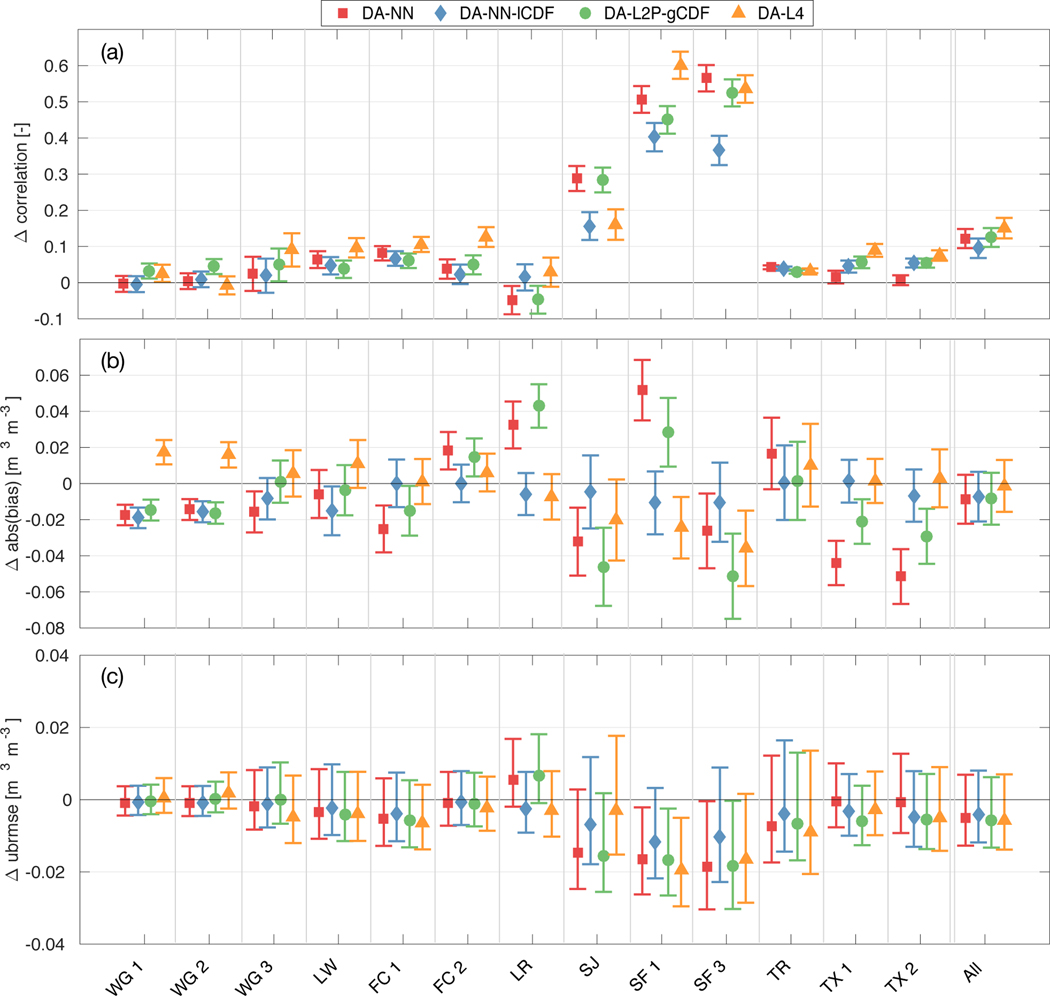
Change in surface soil moisture (a) correlation, (b) absolute bias and (c) ubRMSE versus CVS measurements for the DA-NN (red squares), DA-NN-lCDF (blue diamonds), DA-L2P-gCDF (green circles) experiments and the DA-L4 (orange triangles). Skill changes have been computed against the OL corresponding to each experiment as DA minus OL. Error bars denote the 95% confidence interval. Reference pixel abbreviations are listed in Table 1

Evaluated against the surface CVS measurements (Figure 2), DA-NN and DA-NN-lCDF are able to improve the model skill over the OL. Both experiments yield comparable correlation (Figure 2 (a)) and ubRMSE (Figure 2 (c)) improvements at most reference pixels (exceptions are LR, SF1 and SF3), resulting in similar average correlation increases of 0.12 and 0.10, and ubRMSE reductions of 0.005 m^3^ m^−3^ and 0.004 m^3^ m^−3^ for DA-NN and DA-NN-lCDF. In terms of the bias (Figure 2 (b)), DA-NN generally yields the larger skill changes at individual pixels, including a bias degradation at four pixels, whereas DA-NN-lCDF yields smaller but consistent improvements. On average, this results in a similar bias reduction of 0.009 m^3^ m^−3^ and 0.007 m^3^ m^−3^ for DA-NN and DA-NN-lCDF. The small (albeit not statistically significant at the 5% level) bias reduction for the DA-NN-lCDF estimates with respect to their OL contradicts the intended behavior of the system and might point to issues with the DA system calibration.

**Figure 3.**
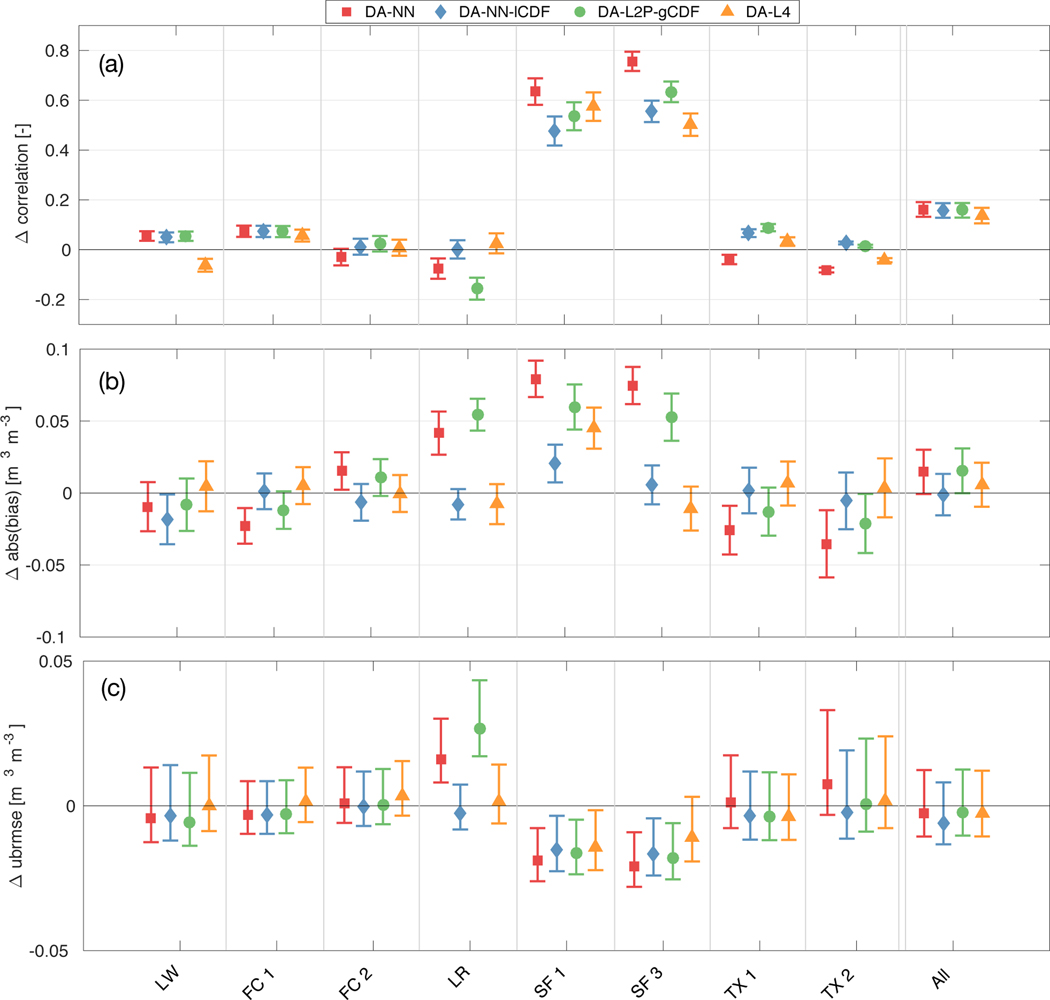
Same as Figure 2, but for the root zone.

Against the CVS root zone measurements (Figure 3), DA-NN-lCDF yields more consistent improvements than DA-NN in terms of the ubRMSE and correlations, but their magnitude is smaller than the less frequent improvements from DA-NN. As a result, the average correlation is improved by 0.16 for both experiments (Figure 3 (a)), but DA-NN-lCDF results in a larger ubRMSE reduction of 0.006 m^3^ m^−3^ compared to 0.003 m^3^ m^−3^ for DA-NN (Figure 3 (c)). In terms of the root zone bias (Figure 3 (b)), both experiments are only able to improve the model skill at approximately half of the reference pixels. The bias degradation at the remaining locations is smaller for the DA-NN-lCDF estimates, resulting in a slight bias reduction of 0.001 m^3^ m^−3^ on average compared to the average bias increase of 0.015 m^3^ m^−3^ for DA-NN.

At many stations, the skill changes with respect to the OL and skill differences between DA-NN and DA-NN-lCDF are small. Notable exceptions are the Little River (LR) and South Fork (SF) watersheds, both of which have previously been identified as sites with large discrepancies between the SMAP retrievals and the in situ measurements [10,49]. At LR, DA-NN consistently degrades the model skill in both soil layers and across all metrics, whereas DA-NN-lCDF yields small or no skill changes. Bearing in mind that the NN retrievals and the OL model estimates have a comparable correlation and ubRMSE skill at LR [25], the results suggest that assimilating the NN retrievals only provides a small amount of novel information to the model, but likely introduces noise that degrades the model skill. For DA-NN-lCDF, the observations appear to have a smaller impact and the soil moisture estimates are less sensitive to retrieval product noise.

At the SF reference pixels, DA-NN improves the soil moisture dynamics, as evident from the significantly (at the 95% confidence level) larger correlation increases and larger (but not statistically significant) ubRMSE reductions in both soil layers compared to DA-NN-lCDF. Figure 1 (d) showed that DA-NN slightly increases the soil moisture variability at SF, likely by introducing the effects of agricultural processes not represented in the model. In contrast, the strong drying in DA-NN at SF (Figure 1 (a)) strongly increases the bias at one surface pixel and at both root zone pixels. Experiment DA-NN-lCDF - by design - only leads to small changes of the bias. This suggests that the observations have a stronger impact in the DA-NN experiment, because more independent satellite information is retained. Therefore, the (reliable) observation information on soil moisture dynamics is used more efficiently in DA-NN. However, the higher impact and the retention of local biases also make the soil moisture estimates more vulnerable to the adverse effects of bias in the retrievals.

**Figure 4.**
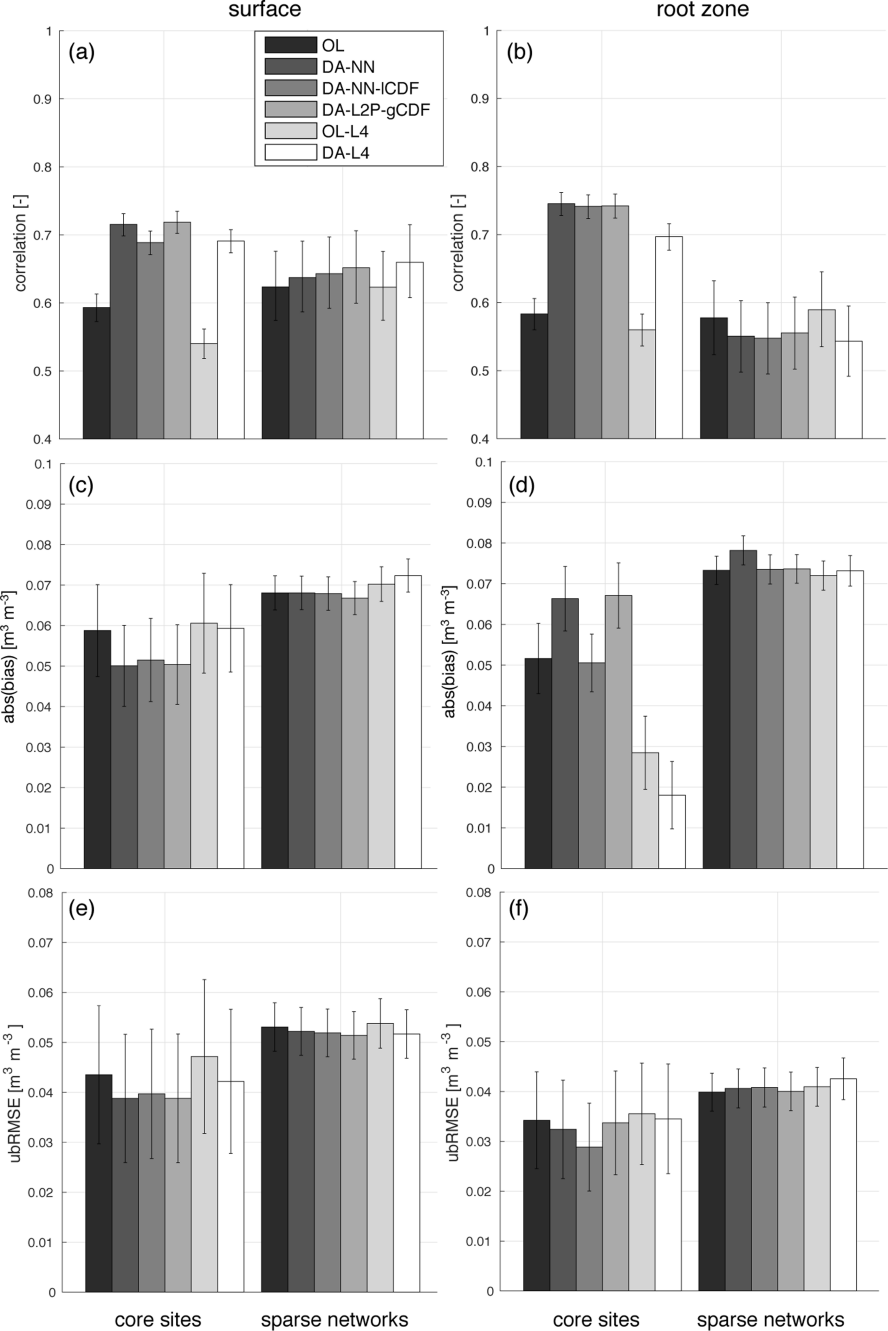
Average metrics for all experiments against core site and sparse network in situ measurements. Shown are (a) the surface correlation, (b) root zone correlation, (c) surface absolute bias, (d) root zone absolute bias, (e) surface ubRMSE, and (f) root zone ubRMSE. The error bars indicate the 95% confidence interval.

When evaluated against sparse network in situ measurements (Figure 4), differences in the average metrics of both experiments are less pronounced than for the CVS evaluation. In the surface layer, both assimilation experiments increase the correlation and reduce the ubRMSE over the OL. For the root zone, both assimilation experiments slightly degrade the model skill compared to the OL for all metrics. However, compared to the error bars, the skill changes observed in the sparse network evaluation are nearly negligible.

#### 4.1.3. Model and observation errors

**Figure 5.**
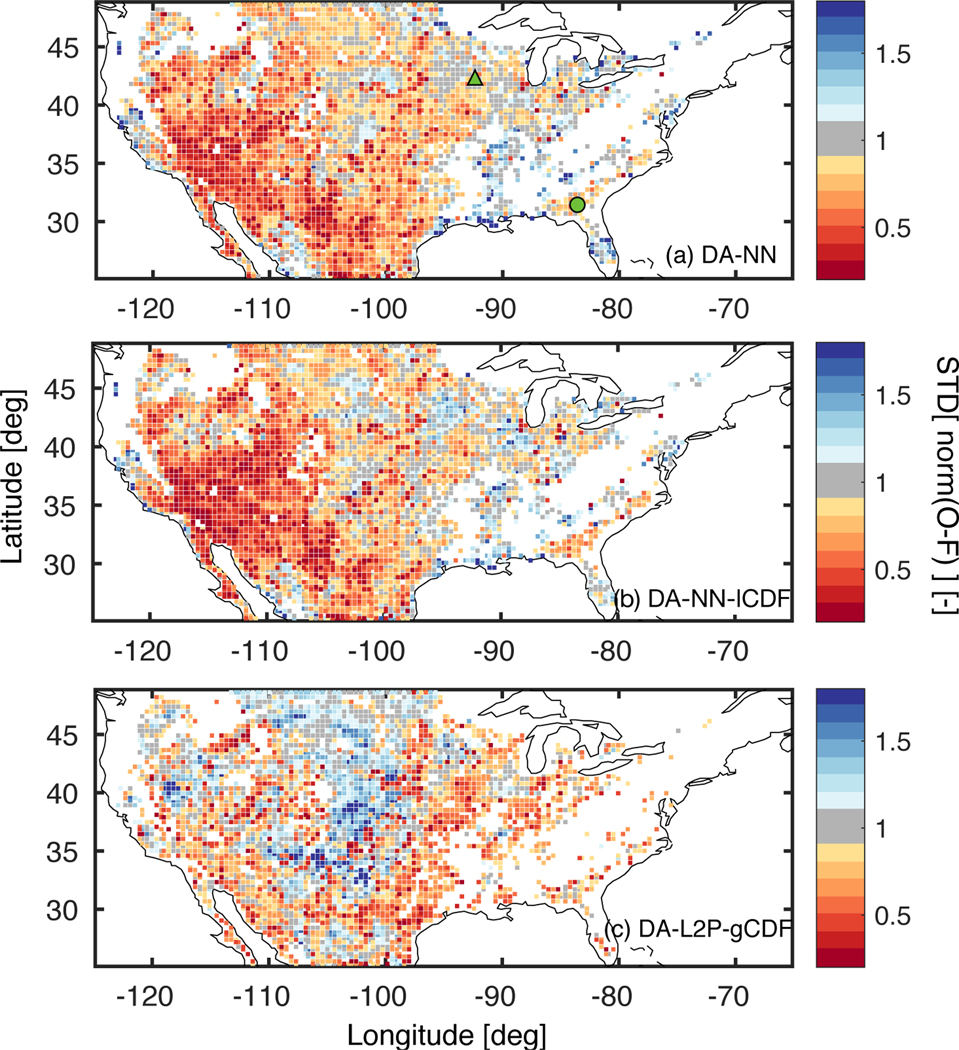
Standard deviation of the normalized innovations (O minus F) for the (a) DA-NN, (b) DA-NN-lCDF and (c) DA-L2P-gCDF experiments. Red colors indicate that the assumed errors are overestimated with respect to the actual errors and blue colors indicate an underestimation. White areas indicate that less than 30 observations were assimilated and no metric was computed. Panel (a) also shows the location of the SF (green triangle) and LR (green circle) sites.

The impact of the assimilated soil moisture observations on the model estimates is driven by (1) the difference between the rescaled observations and the forecast and (2) the relative weight given to the observations and the model during the assimilation. The latter depends on the specified model and observation errors through the Kalman gain. The standard deviation of the normalized observation-forecast differences (Figure 5) shows how accurately the DA system reflects the actual model and observation errors. For both experiments, the DA system tends to overestimate the actual errors (as indicated by values smaller than 1), which is also reflected by the domain average values of 0.89 for DA-NN and 0.68 for DA-NN-lCDF. This more pronounced overestimation for DA-NN-lCDF could be one reason for the apparently smaller observation impact noted above. The inaccurate error characterization could be caused by (1) inaccurate observation errors estimated from the TC analysis (section 2), (2) uncertainties in the model or observation temporal standard deviations used to rescale the observation errors for DA-NN-lCDF or (3) inaccurate model errors - represented by the ensemble spread and driven by the forcing and prognostic perturbations. Points (1) and (3) would affect both assimilation experiments and are thus likely causes for the general error overestimation. Point (2) affects only DA-NN-lCDF and could explain the stronger error overestimation. The model perturbations used here were initially developed for the L4_SM Tb assimilation and yield model standard deviations that might not be appropriate for the soil moisture assimilation conducted here.

#### 4.1.4. Impact on related model fields

The soil moisture skill improvement in DA-NN over OL (with the root zone bias as the only exception) suggests that issues with the retention of local biases (see section 3.3) may in practice be outweighed by the benefit of retaining more of the independent SMAP information. It is important, however, to also assess how the assimilation without local bias correction affects the overland runoff and land evaporation.

**Figure 6.**
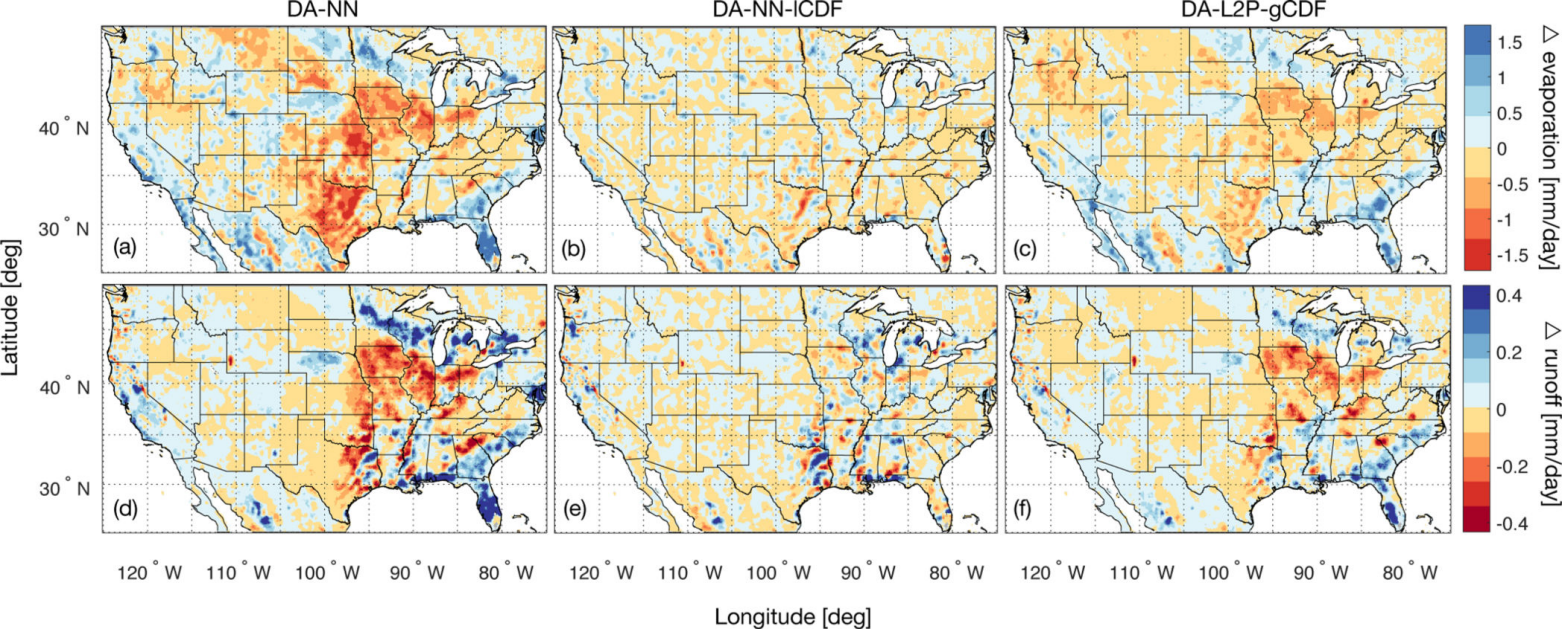
Average land evaporation difference - computed as DA minus OL for the period April 2015 to March 2017 - for the (a) DA-NN, (b) DA-NN-ICDF and (c) DA-L2P-gCDF experiments. Panels (d)-(f) are the same, but for the difference of overland runoff with respect to the OL. Red colors indicate that the assimilation reduces the evaporation and runoff with respect to the OL.

The differences in mean land evaporation for DA-NN and DA-NN-lCDF (Figure 6 (a) and (b)) primarily reflect differences in the mean soil moisture state caused by assimilation of SMAP observations (Figure 1 ). For DA-NN, this includes a reduced evaporation in the region stretching from southeast of the Great Lakes to Texas, for which a strong drying was observed in Figure 1 (a), and an increased evaporation corresponding to the increased soil moisture in Florida. Generally, the land evaporation tends to be more sensitive to soil moisture in the Western US, however, owing to the smaller soil moisture changes introduced there, this increased sensitivity is not evident in the evaporation changes. For the DA-NN-lCDF experiment, the mean soil moisture state is - by design - not changed relative to the OL and as a result no notable changes in the mean land evaporation are introduced by the assimilation.

In terms of the runoff (Figure 6 (d) and (e)), the assimilation mostly introduces changes in regions where the runoff is large, such as the Eastern US and along the West Coast. For DA-NN, these changes mirror the spatial features of the mean soil moisture changes, resulting in a runoff increase in areas with increased soil moisture and vice versa. For DA-NN-lCDF, no notable spatial features were introduced in the mean soil moisture state and thus no spatial features are discernible in the changes to the runoff.

A quantitative validation of the evaporation and runoff changes introduced by DA-NN is difficult due to a lack of reliable reference data. The DA-NN experiment is able to reduce the known evaporation overestimation of the model [50], but the very large changes of ~1 mm/day are likely unrealistic. Furthermore, the runoff reductions introduced by DA-NN intensify the known runoff underestimation of the model [50]. Thus, the soil moisture skill improvements observed for DA-NN do not readily translate into improvements in related water cycle variables. For applications aiming to obtain a comprehensive set of land surface estimates (rather than only improving soil moisture estimates), an additional re-calibration of the soil moisture dependent processes in the land model would be required in order to make the DA-NN approach fully viable.

#### 4.1.5. Discussion of DA-NN and DA-NN-lCDF results

Generally, the DA-NN and DA-NN-lCDF experiments are able to improve the model soil moisture skill over the OL. Particularly over CONUS, where the validation data are dense and where the model generally has a high skill, improving the model through data assimilation is more difficult than in data sparse regions. Additionally, using corrected precipitation forcing data (section 3.1) further limits the skill improvements that can be obtained from an assimilation. The consistent assimilation skill improvements are thus encouraging and demonstrate the great potential of SMAP observations to improve land surface model estimates, in particular in data sparse regions. Remaining differences between the modeled estimates and the in situ measurements are related to uncertainties in the assimilated observations and the model forcing data as well as differences in the ancillary data (for example the soil texture) used in the model and at the ground stations.

In the DA-NN experiment, which retains more of the independent satellite information, the observations have a larger impact on the soil moisture estimates than in the DA-NN-lCDF experiment. When the observation are of high quality and contain novel information, this can lead to larger improvements in the model soil moisture skill than is possible with a local bias correction. However, the larger observation impact also makes the DA-NN more vulnerable to the adverse effects of low-quality satellite observations. This means that the NN assimilation without bias correction can use the observation information more efficiently, but is also less reliable than an assimilation using a localized bias correction. To use the DA-NN approach it is thus crucial to accurately characterize the model and observation errors and to apply a rigorous quality control to the observations. Additionally, to better isolate the reliable retrieval information, it might be beneficial to separately assimilate the different temporal components of the retrievals - i.e., the long-term mean, seasonal, sub-seasonal and interannual signatures [51] - with the DA-NN approach.

### 4.2. Assimilation of NN vs. L2P retrievals

In this section, we compare the assimilation of the NN retrievals (DA-NN) to that of the L2P retrievals (DA-L2P-gCDF) to determine the impact of the different retrieval approaches. In both cases, the global climatology of the observations matches that of the corresponding model estimates.

#### 4.2.1. Mean soil moisture statistics

The spatial patterns of the mean soil moisture differences between DA-L2P-gCDF and OL (Figure 1 (c)) are similar to those observed for the DA-NN experiment (Figure 1 (a)), but generally have a smaller magnitude. Notable discrepancies in the difference spatial patterns of the DA-NN and DA-L2P-gCDF experiments occur along parts of the Rocky Mountains (in Colorado, Wyoming and Idaho), where DA-NN causes a wetting relative to OL, whereas DA-L2P-gCDF introduces mostly small mean soil moisture changes relative to OL. As for DA-NN, the spatial patterns in the mean soil moisture difference between DA-L2P-gCDF and OL reflect the local biases between the L2P retrievals and the model.

The spatial patterns of the standard deviation difference between the DA-L2P-gCDF and OL experiments (Figure 1 (f)) are also very similar to those observed for the DA-NN experiment, but with a slightly smaller magnitude. In addition to the SMAP observations and the ancillary retrieval inputs (VWC and surface temperature), the differences between the L2P retrievals (and corresponding assimilation estimates) and the model are also driven by the ancillary parameter inputs, such as the soil texture. The L2P retrieval algorithm relies on more of these ancillary data than the NN retrievals, and as such the spatial features of the DA-L2P-gCDF estimates correspond less to SMAP observational features than those of the DA-NN estimates.

#### 4.2.2. Evaluation against in situ measurements

Evaluated against the surface CVS measurements (Figure 2), the DA-NN and DA-L2P-gCDF experiments have a very similar skill at most reference pixels and across all metrics. This results in nearly identical average skill improvements for both experiments, with correlation increases of 0.12 and 0.13, bias reductions of 0.009 m^3^ m^−3^ and 0.008 m^3^ m^−3^, and ubRMSE reductions of 0.005 m^3^ m^−3^ and 0.006 m^3^ m^−3^ for DA-NN and DA-L2P-gCDF, respectively.

Similarly, the skill of the DA-NN and DA-L2P-gCDF estimates against the root zone CVS measurements (Figure 3) is nearly identical at most reference pixels. This is also reflected in the average correlation improvements of 0.16 and ubRMSE reductions of 0.003 m^3^ m^−3^ for both experiments. Both assimilations are only able to reduce the root zone bias at about half of the reference pixels and the relatively large bias degradation at the remaining pixels results in an average bias increase of 0.015 m^3^ m^−3^ and 0.016 m^3^ m^−3^ for DA-NN and DA-L2P-gCDF.

As before, the LR and SF watersheds show more pronounced differences between the two assimilation experiments. At SF, DA-NN generally obtains larger correlation improvements than DA-L2P-gCDF in both soil layers, but DA-L2P-gCDF leads to smaller bias degradations (or larger bias reductions). Given that the NN and L2P retrievals have a similar skill at the SF pixels [25], the results suggest that the observations have a larger impact on the analysis for DA-NN than for DA-L2P-gCDF.

At LR, DA-L2P-gCDF shows the same consistent skill degradation as DA-NN, but the magnitude of the degradation is larger. Previously, *Kolassa et al.* [25] found that the L2P retrievals had a significantly better (at the 95% confidence level) correlation skill than the NN retrievals and the model at LR, indicating that at LR the L2P retrievals capture soil moisture information that is not represented in the other products. The DA-L2P-gCDF skill degradations thus suggest that at LR, the DA system is either not able to extract this independent information or is too sensitive to potential noise in the retrievals.

Against the sparse network measurements (Figure 4), the DA-NN and DA-L2P-gCDF experiments have nearly identical correlation and ubRMSE skill in both soil layers. In terms of the bias, DA-L2P-gCDF is able to slightly reduce the bias in the surface and root zone layers, whereas DA-NN slightly increases the surface bias against the sparse network measurements.

#### 4.2.3. Model and observation errors

The specified model and observation errors of the DA-L2P-gCDF experiment (Figure 5(c) ) underestimate the actual errors in some regions, particularly in the Central US. This is reflected in the higher domain average value of 1.01 for DA-L2P-gCDF compared to 0.89 for DA-NN. These differences can be caused by (1) different errors for the L2P retrievals compared to the NN retrievals generated with the TC analysis (section 2.1.2) and (2) the rescaling of the L2P errors in the DA-L2P-gCDF experiment with the ratio of the global standard deviations of the model and observations (section 2.1.2).

#### 4.2.4. Impact on related model fields

The impact of the DA-L2P-gCDF assimilation on the modeled land evaporation (Figure 6 (c)) has similar spatial patterns as the impact of the DA-NN assimilation and primarily reflects the changes in the mean soil moisture state. Generally, the magnitude of the evaporation changes is smaller for the DA-L2P-gCDF estimates because of the smaller impact of DA-L2P-gCDF on the mean soil moisture state compared to DA-NN.

Similarly, the spatial patterns of the overland runoff changes introduced by DA-L2P-gCDF (Figure 6 (f)) are very similar to those introduced by DA-NN, but have a smaller magnitude as a result of the smaller soil moisture impact in DA-L2P-gCDF compared to DA-NN. The larger differences between the mean soil moisture state of DA-NN and DA-L2P-gCDF near the Rocky Mountains are not propagated into the runoff, as a result of the reduced runoff sensitivity to soil moisture in areas where the runoff magnitude is small (see also section 4.1.4).

#### 4.2.5. Discussion of DA-NN and DA-L2P-gCDF results

Overall, the skill of the DA-NN and DA-L2P-gCDF experiments is very similar, suggesting that a global CDF-matching of an existing soil moisture retrieval product can yield a comparable soil moisture skill when a retrieval in the model climatology is not possible. Additionally, the skill differences between the DA-NN and DA-L2P-gCDF experiments are related to (1) differences in the retrieval product skill and (2) differences in the amount of novel information that each retrieval product provides to the model. The skill of both retrieval products was extensively evaluated against in situ measurements in [25], who found it to be comparable with somewhat better correlations for the L2P retrievals and a lower ubRMSE for the NN retrievals. Our findings suggest that the impact of these retrieval skill differences on modeled soil moisture estimates generated here is negligible.

The amount of novel information that each data product provides to the model is more difficult to quantify. As a proxy, we compared the model skill from the DA-NN-lCDF experiment to the model skill from an assimilation of the L2P retrievals with a local CDF-matching (DA-L2P-CDF; not shown here). Since the bias correction and assimilation setup in both experiments are the same, differences in the resulting model skill are related to differences in the retrieval skill (which are small, see above) and differences in the independent information provided by both products. The DA-NN-lCDF and DA-L2P-CDF experiments were found to have a nearly identical average surface correlation against the core site measurements of 0.69 for both experiments, and a similar average absolute bias of 0.052 m^3^ m^−3^ and 0.051 m^3^ m^−3^ for DA-NN-lCDF and DA-L2P-CDF, respectively. This suggests that the amount of independent information provided by each retrieval product is comparable.

### 4.3. Assimilation of soil moisture vs. brightness temperatures

Finally, we evaluate the skill improvements from the soil moisture assimilation experiments presented in the previous sections against those obtained from the brightness temperature assimilation implemented in the SMAP L4_SM system. The L4_SM system has been extensively tested and validated [18,52] and thus the skill of the L4_SM estimates can be considered as somewhat of a baseline for the amount of information that a DA system can extract from the SMAP observations. To some extent, the comparison with the L4_SM estimates also assesses the feasibility of the NN as a tool to project SMAP Tb into the modeled soil moisture space, which is similar to the projection of modeled soil moisture estimates into the SMAP Tb space by the L4_SM RTM (although bearing in mind that the Tb observations are locally rescaled in the L4_SM system). As before, we focus on comparing skill improvements to account for the fact that the soil moisture assimilation experiments and the L4_SM estimates have a slightly different OL (section 2.1.3).

Evaluated against the surface CVS measurements (Figure 2), the L4_SM estimates are able to yield higher correlation improvements than the soil moisture assimilation experiments at most stations, resulting in the largest average correlation improvement of 0.15. In terms of the ubRMSE, the skill improvements of the Tb and soil moisture assimilations are similar with an average ubRMSE reduction of 0.006 m^3^ m^- 3^ for DA-L4. Like the DA-NN and DA-L2P-gCDF experiments, DA-L4 leads to a surface bias degradation at several stations. However, these are smaller in magnitude than for DA-NN and DA-L2P-gCDF and are balanced by bias improvements, for example at the SF reference pixels. As a result, DA-L4 behaves as designed and does not significantly change the average bias with respect to its OL.

Against the root zone CVS measurements (Figure 3), DA-L4 has the lowest average correlation skill improvement of 0.14. The average ubRMSE reduction for DA-L4 of 0.003 m^3^ m^−3^ is similar to the reductions obtained from the soil moisture assimilation experiments. In terms of the bias, the DA-L4 estimates behave as intended and only slightly change the bias relative to the OL at most reference pixels (an exception is the SF1 pixel). The resulting average bias increase of 0.006 m^3^ m^−3^ is small compared to the values for DA-NN and DA-L2P-gCDF, but slightly larger than the bias reduction of 0.001 m^3^ m^−3^ obtained with DA-NN-lCDF.

The DA-L4 and DA-NN-lCDF experiments are the most similar in terms of the observation rescaling applied prior to the assimilation (although different moments are rescaled locally in each case), but this is not necessarily reflected in a more comparable skill of both experiments. This is partly due to differences introduced by the retrieval algorithm, but *De Lannoy and Reichle* [17] also showed that the assimilation of locally rescaled SMOS Tbs or soil moisture estimates extracted very different information from the observations locally. Thus, it is not surprising that DA-NN-lCDF and DA-L4 have different skills at individual reference pixels.

Similarities between the DA-L4 and DA-NN-lCDF experiments exist at the LR reference pixel, where both experiments generally improve the model skill, whereas the two experiments using a global observation rescaling (DA-NN and DA-L2P-gCDF) consistently degrade the model skill. These differences are not related to the retrieval product skill and could thus be related to (1) a higher level of retained observation noise or (2) an uncertain error characterization in the DA-NN and DA-L2P-gCDF experiments.

Evaluated against the sparse network measurements (Figure 4), the skill differences of DA-L4 relative to its OL are very small and consistently within error bars. The Tb assimilation slightly improves the correlation and ubRMSE skill in the surface layer, but slightly degrades the skill in the root zone. For the bias, the behavior is inverted, with a slight bias improvement in the root zone.

Overall, the soil moisture assimilation experiments and DA-L4 are able to achieve very similar skill improvements over their respective open loops. This supports the finding of *De Lannoy and Reichle* [17] that the assimilation of SMOS Tbs and soil moisture estimates - while locally different - resulted in model estimates with a comparable average skill against in situ measurements. Taken together, the results suggest that the NN method could be a viable assimilation alternative when a Tb assimilation is not possible (e.g., due to issues with the RTM calibration or a too high complexity of the RTM).

Furthermore, the assimilation configuration used in the experiments here is very close to that of the SMAP L4_SM system and as such might not represent the optimal configuration for soil moisture retrieval assimilation. A better calibration of the model perturbations might further improve the observation impact and increase the skill improvements from the soil moisture assimilation experiments.

## 5. Conclusions and Perspectives

In this study we compared different methods to extract soil moisture information from SMAP observations through data assimilation. In particular, we focused on the potential of NN techniques to reduce the need for bias correction prior to an assimilation in order to maximize the amount of independent satellite information that is used to inform the model. We conducted three experiments to assimilate SMAP soil moisture retrievals into the NASA CLSM and evaluated the resulting soil moisture estimates against in situ measurements from the SMAP core validation sites as well as two sparse networks. For reference, we also compared our soil moisture assimilation experiments against the skill of the SMAP L4_SM estimates generated through a SMAP Tb assimilation.

All of the SMAP data assimilation experiments included in our study were generally able to improve the surface and root zone soil moisture model skill over the respective open loop (model run without data assimilation) when evaluated against the CVS in situ measurements (with the exception of the root zone bias). This demonstrates the general potential of the SMAP observations to inform the model, irrespective of the data assimilation approach chosen, and confirms previous findings [18,52]. For most reference pixels, the improvements over the OL were small and differences in the average metrics were mostly driven by a few pixels with large improvements. However, the improvement over the model skill in data-rich region such as the US is limited because the model skill is generally high. Larger improvements in the model skill can be expected in data-sparse regions. Measurements at the sparse network sites are less representative of the grid-cell scale estimates from the model and retrievals. Moreover, the sparse networks include many stations where microwave-based soil moisture retrievals are not reliable. Therefore, the skill improvements over the open loop were generally smaller or sometimes negative for the sparse networks.

Comparing the three soil moisture assimilation experiments showed that using a global observation rescaling (DA-NN and DA-L2P-gCDF) better retained the independent soil moisture information provided by the SMAP retrievals and led to a larger impact of the observations during the assimilation. This resulted in larger soil moisture skill improvements at many reference pixels compared to the improvements obtained when using a local rescaling (DA-NN-lCDF). However, it also made the soil moisture estimates more sensitive to a skill degradation in locations where the observations were uncertain. On average, the assimilation resulted in slightly higher skill improvements against the surface in situ measurements for DA-NN and DA-L2P-gCDF and slightly higher skill improvements in the root zone for DA-NN-lCDF. Overall, the results suggest that the global rescaling approaches could potentially be very beneficial for soil moisture estimation under the condition of (1) a good observation error characterization, (2) a rigorous observation quality control and (3) a potential component-wise assimilation [51] to better isolate the reliable satellite information.

The experiments using global observation rescaling introduced large changes in the land evaporation and runoff that were likely unrealistic in magnitude. This showed that using the NN assimilation method for purposes other than improving soil moisture estimates is not recommended without a careful re-calibration of the model processes translating soil moisture changes into changes of other model variables.

Instead of assimilating the NN retrievals without further bias correction (in DA-NN), similar results were obtained when assimilating physically-based retrievals after a global bias correction (in DA-L2P-gCDF). We previously showed that the retrieval product skill and amount of independent information provided by the NN and L2P retrievals is comparable and thus the similar skill of the DA-NN and DA-L2P-gCDF estimates indicates that the two rescaling methods are approximately equivalent. However, the relatively short record length of the SMAP observations implies that sampling errors impact both the NN method and the two CDF-matching approaches used here and that the results might change as longer data records become available.

Finally, compared to the skill improvements obtained from the SMAP Tb assimilation implemented in the SMAP L4_SM system, the soil moisture assimilation experiments had comparable average correlation and ubRMSE skill. Differences in the average bias changes between the L4_SM estimates and the experiments using global observation rescaling exist as a result of the local Tb rescaling implemented in the L4_SM system. Overall, the results suggest that on average there is no particular advantage to assimilating either Tbs or soil moisture estimates, although locally the choice could result in statistically significant skill differences.

## Figures and Tables

**Table 1.** Overview of the SMAP Calibration/Validation core sites. Shown are the site name, site key, reference pixel ID (RPID), location, climate, land cover and the availability of root zone measurements (from left to right). The measurement depth for surface soil moisture is 5 cm at all sites. The measurement depth for root zone soil moisture ranges from 30 cm to 75 cm depending on the station [18]

Site	site key	RPID	US state	climate	land cover	root zone
Walnut Gulch	WG1	16010906	Arizona	arid	shrub open	no
	WG2	16010907				no
	WG3	16010913				no

Little Washita	LW	16020907	Oklahoma	temperate	croplands and pasture	yes

Fort Cobb	FC1	16030911	Oklahoma	temperate	croplands and pasture	yes
	FC2	16030916				yes

Little River	LR	16040901	Georgia	temperate	croplands / natural mosaic	yes

St. Joseph’s	SJ	16060907	Indiana	cold	croplands	no

South Fork	SF1	16070909	Iowa	cold	croplands	yes
	SF2	16070910				no
	SF3	16070911				yes

Tonzi Ranch	TR	25010911	California	temperate	woody savannas	no

TxSON	TX1	48010902	Texas	temperate	grasslands	yes
	TX2	48010911				yes

**Table 2.** Ensemble perturbations applied to the forcing variables - precipitation (P), downward shortwave (DSW) radiation and downward long wave (DLW) radiation - and to the Catchment model prognostic variables - surface excess (srfexc) and catchment deficit (catdef). Shown are the perturbation type, which is either multiplicative (M) sampled from a log-normal distribution or additive (A) sampled from a normal distribution, the perturbation standard deviation (std dev), the temporal and spatial correlation lengths as well as the cross-correlations of the forcing variables. Perturbations to the prognostic variables are not cross-correlated.

	type	std dev	temporal correlation	spatial correlation	cross correlation with		
					P	DSW	DLW
P	M	0.5	24 h	0.5 deg	-	−0.8	0.5
DSW	M	0.3	24 h	0.5 deg	−0.8	-	−0.5
DLW	A	20 W m^−2^	24 h	0.5 deg	0.5	−0.5	-
srfexc	A	0.24 kg m^−2^ h^−1^	3 h	0.3 deg			
catdef	A	0.16 kg m^−2^ h^−1^	3 h	0.3 deg			

**Table 3.** Overview of the soil moisture (SM) model and data assimilation experiments.

Experiment Name	Observations assimilated	Bias correction	Model configuration
OL	none	n/a	Nature Run v5
DA-NN	SMAP NN SM	n/a*	Nature Run v5
DA-NN-lCDF	SMAP NN SM	local CDF-matching	Nature Run v5
DA-L2P-gCDF	SMAP L2P SM	global CDF-matching	Nature Run v5
OL-L4	None	n/a	Nature Run v4
DA-L4	SMAP Tb	seasonal climatology matching	Nature Run v4

* global bias correction implicit
